# Global distribution of animal sporotrichosis: A systematic review of Sporothrix sp. identified using molecular tools

**DOI:** 10.1016/j.crmicr.2022.100140

**Published:** 2022-05-19

**Authors:** Debora Salgado Morgado, Rodolfo Castro, Marcelo Ribeiro-Alves, Danielly Corrêa-Moreira, Julio Castro-Alves, Sandro Antonio Pereira, Rodrigo Caldas Menezes, Manoel Marques Evangelista Oliveira

**Affiliations:** a Laboratory of Clinical Research on Dermatozoonoses in Domestic Animals, Evandro Chagas National Institute of Infectious Diseases, Oswaldo Cruz Foundation, Rio de Janeiro, Brazil; b Department of Education, Evandro Chagas National Institute of Infectious Diseases, Oswaldo Cruz Foundation, Rio de Janeiro, Brazil; c Sergio Arouca National School of Public Health, Oswaldo Cruz Foundation, Rio de Janeiro, Brazil; d Institute of Collective Health, Federal University of the State of Rio de Janeiro, Brazil; e Laboratory of AIDS and Molecular Immunology, Oswaldo Cruz Institute, Oswaldo Cruz Foundation, Rio de Janeiro, Brazil; f Laboratory of Taxonomy, Biochemistry and Bioprospecting of Fungi, Oswaldo Cruz Institute, Oswaldo Cruz Foundation, Rio de Janeiro, Brazil; g Clinical Research Platform, FIOCRUZ, Rio de Janeiro, Brazil; h Evandro Chagas National Institute of Infectious Diseases, Oswaldo Cruz Foundation, Rio de Janeiro, Brazil; i International Platform for Science, Technology and Innovation in Health - PICTIS

**Keywords:** Sporothrix, Sporotrichosis, Cat diseases, Dog diseases, Systematic review

## Abstract

Sporotrichosis is a subcutaneous mycosis caused by thermodimorphic fungi of the genus Sporothrix. The phenotypic and genotypic differences of the isolates within the genus Sporothrix have been associated with their geographic distribution, virulence capacity, or clinical manifestation of sporotrichosis. Therefore, it is crucial to identify the causative agent of sporotrichosis. However, there are few case reports and studies in animals compared to those in humans, despite the substantial increase in the number of cases of sporotrichosis by zoonotic transmission, especially in endemic areas. Considering the epidemiological importance, taxonomic evolution and worldwide distribution of these fungi in the last decade, there is interest in identifying the species of the genus Sporothrix in different regions of the world. This study aimed to analyze the geographic distribution of animal sporotrichosis in the world, caused by pathogenic species identified by use of molecular tools. This systematic review of articles from 2007 to 2021 analyzed the geographic distribution of species that cause sporotrichosis in cats, dogs and other animals. It demonstrated that the most identified species were S. brasiliensis, isolated from cats in Brazil and S. schenckii isolated from cats in Malaysia. We show the lack of studies in global areas and reinforce the need to use molecular tools to identify and monitor potential pathogens.

## Highlights

• First systematic review that reports exclusively the geographic distribution of animal sporotrichosis in the world, focusing in molecular identification of these species.
• Scarcity of epidemiological studies in global areas.
• The importance of apply molecular tools to identify and monitor potential pathogens to improve one health concept.

## Graphical abstract

**Figure:**
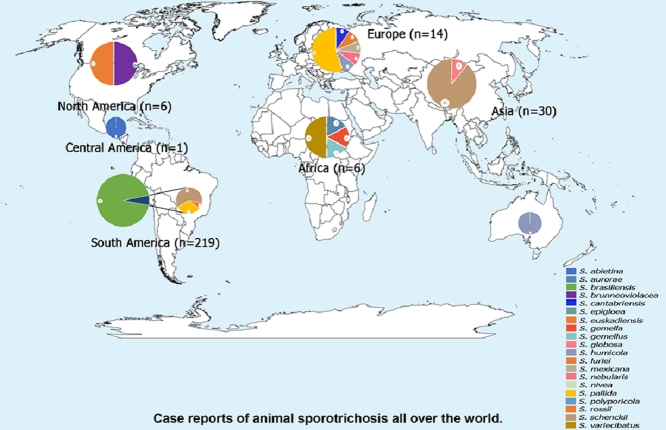

## 1 Introduction

Sporotrichosis is a subcutaneous mycosis caused by thermodimorphic fungi of the Sporothrix genus. This fungal infection is globally distributed. However, the actual incidence of the disease is difficult to measure, since sporotrichosis is not a notifiable disease in most countries (Gremião et al., 2015). The “classical” transmission of the etiologic agent occurs through the skin by traumatic inoculation of the fungus present in vegetal, soil or organic matter containing Sporothrix sp conidia (Schubach et al., 2004).

**Table:** 

Taxonomy: Eukaryota; Opisthokonta; Fungi; Dikarya; Ascomycota; saccharomyceta; Pezizomycotina; leotiomyceta; sordariomyceta; Sordariomycetes; Sordariomycetidae; Ophiostomatales; Ophiostomataceae. **Sporothrix** Hektoen & Perkins (1900) Synonyms: Sporotrichopsis Gueguen. De Beurmann and Gougerot (1911). [type species S. beurmannii; nom. inval., Art. 38.1] Dolichoascus Ansel and Thibaut (1970). [type species D. schenckii; nom. inval., Art. 40.1] Sporothrix section Sporothrix Weijman and de Hoog (1985).

According to De Beer et al. (2016), the Sporothrix genus is worldwide distributed and it is divided into two clades: The clinical or pathogenic clade composed of S. brasiliensis, S. schenckii, S. globosa, and S. luriei (former S. schenckii var. luriei) and the environmental clade, composed by S. pallida complex (S. chilensis, S. mexicana, S. humicola, and S. pallida former S. albicans) and the S. stenoceras complex.

The state of Rio de Janeiro, Brazil, has been experiencing a particular situation since 1998. A hyperendemic sporotrichosis, in which it was observed that the transmission of the fungus to man did not occur in a classical way, but was transmitted zoonotically, through scratching, biting or contact with exudates from skin lesions of infected cats (Gremião et al., 2017).

S. brasiliensis, S. schenckii, and Sporothrix humicola are considered causal agents of feline sporotrichosis, and the distribution of cases is wide, reaching all continents, according to the few studies published to date (Kano et al., 2015a; Rodrigues et al., 2016; Siew, 2017; Boechat et al., 2018; Duangkaew et al., 2018; Makri et al., 2020; Rodrigues et al., 2020).

Similarly, to humans, dogs have three clinical forms of the disease: localized cutaneous, lymphocutaneous, and disseminated form (Crothers et al., 2009; Boechat et al., 2021). However, canine sporotrichosis is rare, with scarce case reports (Viana et al., 2018).

For the molecular characterization of the species, the extraction, amplification and sequencing of the DNA of the isolates are used by employing the polymerase chain reaction (PCR) (Marimon et al., 2007; Rodrigues et al., 2013a). Phylogenetic analysis of Sporothrix species has traditionally been performed using sequencing data from single or multiple conserved genes, mainly the chitin synthase (CHS), β-tubulin and calmodulin gene (CAL). The latter is the reference standard for the molecular identification of species of the genus Sporothrix (Marimon et al., 2007; New et al., 2019). Phenotypic tests alone are not sufficient to identify species of the genus Sporothrix, due to the uncertainty of the tests, which require the use of molecular methodologies (Oliveira et al., 2011b).

It is important to note that fungal infections are often neglected (Seyedmousavi et al., 2015), and public health policies and strategic plans to prioritize these infections are lacking. Several reports have shown alarming concern about the occurrence of cases of zoonotic sporotrichosis in non-endemic regions, such as the case of animal sporotrichosis by S. brasiliensis in Argentina, due to a potential transboundary expansion of the species (Gremião et al., 2020). It is important to highlight that many studies have identified more than one species within the same endemic area (Oliveira et al., 2011a, 2011b) and that some studies in murine models have shown differences in the virulence potential among the main pathogenic species of the genus Sporothrix (Arrillaga-Moncrieff et al., 2009; Corrêa-Moreira et al., 2021). Therefore, the interest in identifying species of the genus Sporothrix in different regions of the world has increased, due to their epidemiological importance, taxonomic evolution and geographic distribution (Chakrabarti et al., 2015). Based on these data, this study aimed to analyze the worldwide distribution of the etiologic agents of sporotrichosis in cats, dogs and other animals, identified by molecular tools.

## 2 Methods

### 2.1 Search activities and screening process

Five bibliographic databases (PubMed, Web of Science, Lilacs, Medline, and Scopus) were searched. Following the Preferred Reporting Items for Systematic Reviews and Meta‐Analyses (PRISMA) statement, consulted at http://www.prisma-statement.org, two independent reviewers screened titles and abstracts after excluding repeated publications. The eligibility criteria followed to include articles were as follows: (a) articles in English; (b) articles from 2007 to 2021; (c) all articles had to identify animal sporotrichosis, including dogs, cats, and other animals such as tiger-quoll, insects, equine, and naturally infected mice; (d) species identification was required, however, location was not mandatory. The isolates described as “not known” were analyzed and reported as unknown. The exclusion criteria used were: non-inclusion of the theses, dissertations, monographs or publications without strain identification (without verification code), experimental model, human and environmental isolates, and unavailable full texts.

The year 2007 was chosen to initiate the analysis, as a consequence of the description of seven new pathogenic species of Sporothrix, based on molecular and phenotypic studies that demonstrated intraspecific variability among isolates morphologically identified as S. schenckii. This indicates that it should not be considered a single species causing sporotrichosis, but rather a complex of species.

### 2.2 Data extraction and epidemiological analysis

Two reviewers independently extracted the following variables: identified strain number; country of origin; city of origin (not obligatory); species identification; clinical or environmental clade; and strain of origin. Data analysis was conducted in the R environment version 4.1.2.

## 3 Results

### 3.1 Study selection process

**Fig. 1:**
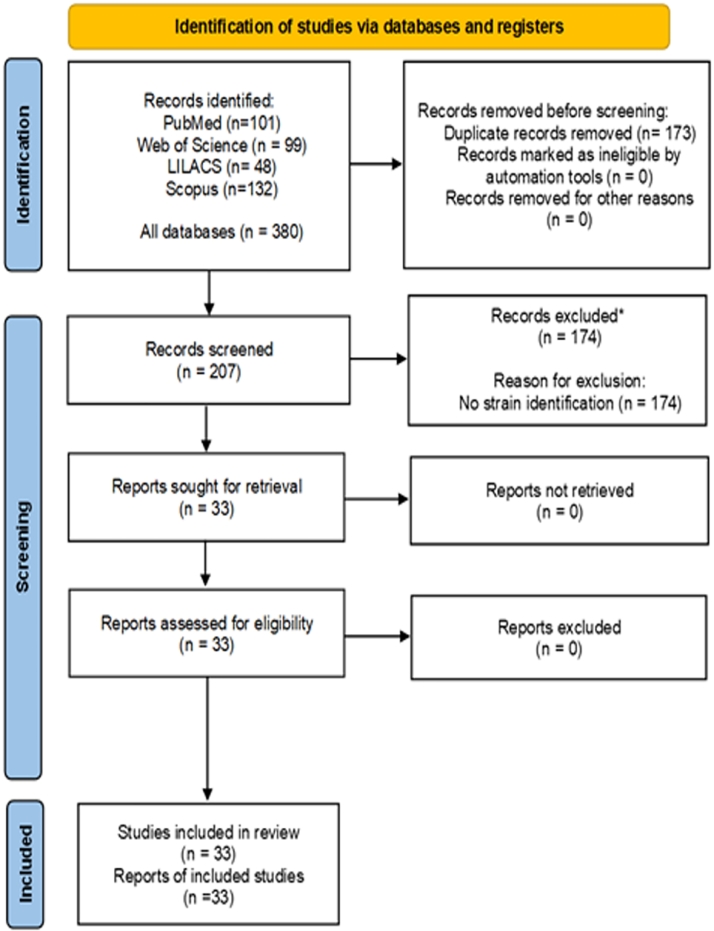
PRISMA 2020, flow diagram of the search and inclusion process in the study.

Fig. 1 shows the flowchart of the study selection process. A total of 380 articles were retrieved from the five databases; After excluding repeated publications, 207 articles were selected by evaluating the full-text, and finally a total of 33 articles were included for analysis.

### 3.2 Distribution of species by continent

**Fig. 2:**
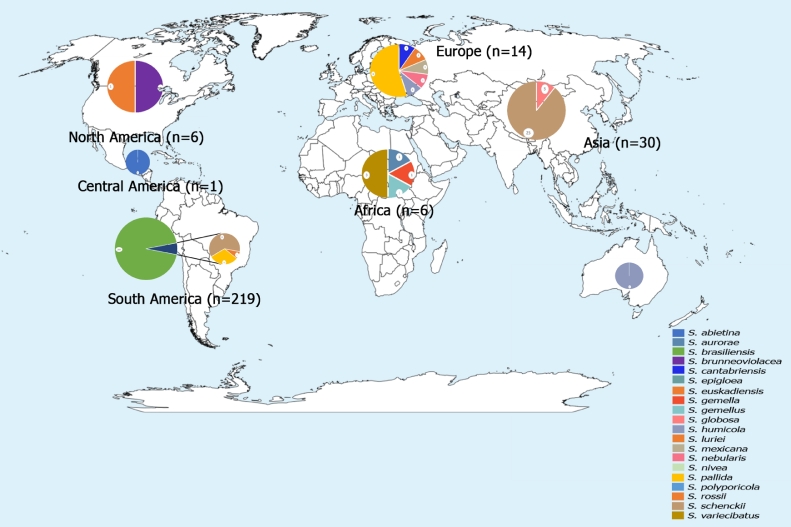
Case reports of animal sporotrichosis all over the world. The sizes of the graphs represent quantitative differences of the cases, in highest number in South America, followed by Asia and Europe. North America and Africa reported the same number of cases and Central America was the continent with fewest reported cases. Only one case was reported in Oceania.

Fig. 2 shows the distribution of each isolate by continent. South America was the continent where the highest number of cases of animal sporotrichosis was reported, followed by Asia and Europe. North America and Africa reported a similar number of cases. Central America and Oceania reported the same number of cases.

#### 3.2.1 South America

A total of 216 isolates of Sporothrix sp. were reported from two South American countries: Brazil and Argentina. The South American continent was the first in number of sporotrichosis cases identified in the study. Most isolates were identified in the study from Brazil, cats (158 isolates) and dogs (52 isolates). The most prevalent species on the continent was S. brasiliensis (199 isolates), followed by S. schenckii (6 isolates). In Argentina 4 isolates S. brasiliensis and 2 isolates S. schenckii were identified and isolated from cats and other animals (equine and mouse), respectively. For species identification, the most used molecular method was the CAL gene (55%), followed by T3B fingerprinting (44%), ITS region (7%), β-tubulin gene (5%), RFLP-CAL (Restriction Fragment Length Polymorphism- Calmodulin gene) (2%), and CHS gene (1%) (Table 1).

**Table 1:** Sporothrix species identified by different molecular methods and described all over the world. Absolute number and percentages of the species determined by each technique are demonstrated.

**Country**	**Species**	**Clade**	**Molecular Method**							**Animal**				
				
			β-**Tub**	**CAL**	**CHS**	**ITS**	**T3B**	**RFLP**	**Others**	**Cat**		**Dog**		**Other**

USA	S. brunneoviolacea	ENV	2(100%)	1(50%))	0(0%)	2(100%)	0(0%)	0(0%)	1(50%)	0(0%)		0(0%)		1(50%)
	S. rossii									0(0%)		0(0%)		1(50%)

Mexico	S. abietina	ENV	1(100%) %)	1(100%)	0(0%)	1(100%)	0(0%)	0(0%)	0(0%)	0(0%)		0(0%)		1(100%)
Argentina	S. brasiliensis	CLI								4(67%)		0(0%)		0(0%)
	S. schenckii	CLI	0(0%)	6(100%)	0(0%)	0(0%)	0(0%)	0(0%)	2(33%)	0(0%)		0(0%)		2(33%)

Brasil	S. brasiliensis	CLI								150(71%)		49(23%)		0(0%)
	S. luriei	ENV								0(0%)		1(0.47%)		0(0%)
	S. pallida	ENV	11(5%)	114(54%))	3(1%)	15(7%)	92(44%)	4(2%)	72(34%)	4(2%)		0(0%)		0(0%)
	S. schenckii	CLI								4(2%)		2(0.94%)		0(0%)
Sweden	Ophiostoma stenoceras	ENV	1(100%)	1(100%)	0(0%)	1(100%)	0(0%)	0(0%)	1(100%)	0(0%)		0(0%)		1(100%)
Spain	S. cantabriensis	ENV									0(0%)		0(0%)	1(33%)
	S. euskadiensis	ENV	3(100%)	3(100%)	0(0%)	3(100%)	0(0%)	0(0%)	1(33%)		0(0%)	0(0%)		1(33%)
	S. nebularis	ENV									0(0%)	0(0%)		1(33%)

Italy	S. mexicana	ENV	0(0%)	1(100%)	0(0%)	1(100%)	0(0%)	0(0%)	1(100%)		0(0%)	1(100%)		0(0%)
Germany	S. pallida	ENV	1(17%)	6(100%)	2(33%)	1(17%)	0(0%)	0(0%)	3(50%)	0(0%)		0(0%)		6(100%)
UK	S. humicola	ENV	1(100%)	1(100%)	0(0%)	0(0%)	0(0%)	0(0%)	0(0%)	1(100%)		0(0%)		0(0%)
South Africa	S. aurorae	ENV								0(0%)		0(0%)		1(17%)
	S. gemella	ENV								0(0%)		0(0%)		1(17%)
	S. gemellus	ENV	4(67%)	3(50%)	0(0%)	6(100%)	0(0%)	0(0%)	4(67%)	0(0%)		0(0%)		1(17%)
	S. variecibatus	ENV								0(0%)		0(0%)		3(50%)
Malaysia	S. schenckii	CLI	0(0%)	25(100%)	0(0%)	18(72%)	1(4%)	0(0%)	18(72%)	25(100%)		0(0%)		0(0%)
Japan	S.globosa	CLI	0(0%)	3(100%)	0(0%)	1(33%)	0(0%)	0(0%)	2(67%)	3(100%)		0(0%)		0(0%)
Tasmania	S. humicola	CLI	1(100%)	1(100%)	0(0%)	1(100%)	0(0%)	0(0%)	0(0%)	0(0%)		0(0%)		1(100%)

β-Tub – Beta tubulin gene; CAL – Calmodulin gene; CHS - chitin synthase gene; ITS – Internal transcribed spacer; T3B – T3B fingerprinting; RFLP - Restriction Fragment Length Polymorphism

#### 3.2.2 Asia

A total of 28 isolates were described in Japan and Malaysia. In Malaysia, 25 isolates of S. schenckii, and in Japan 3 isolates S. globosa from the clinical clade of cats were identified. For species, the most commonly used molecular method was the PCR with the sequencing of CAL gene (100%), followed by ITS region (68%), and other molecular methods (71%) (Table 1).

#### 3.2.3 Europe

The total number of Sporothrix sp. species reported in Europe was 12 isolates. Germany was the country with the highest number of isolates with six strains identified, followed by Italy (1 isolate), Spain (3 isolates), Sweden (1 isolate), and the United Kingdom (1 isolate). Only Italy isolated samples from dogs, the United Kingdom isolated cat samples, and other countries obtained samples from insects. All countries isolated species from the environmental clade: S. cantabriensis, S. euskadiensis, S. mexicana, S. nebularis, S. pallida, S. humicola and Ophiostoma stenoceras. For species identification, the most used molecular method was the CAL gene (100%), followed by ITS region, β-tubulin gene (50%), and CHS gene (17%) (Table 1).

#### 3.2.4 North America

In the United States, two isolates of Sporothrix sp. were reported from the environmental clade (S. brunneovilacea and S. rossii). The isolates were obtained from insects. Molecular methods, PCR with the ITS region and β-tubulin gene, (100%), CAL gene and other molecular methods (50%) were used to identify the species (Table 1).

#### 3.2.5 Africa

In South Africa, six isolates of Sporothrix sp. from the environmental clade (S. aurorae, S. gemella, S. gemellus and S. variecibatus), were identified from insects. For species identification, the most commonly used molecular method was PCR followed by the ITS region (100%), β-tubulin gene, and other molecular methods (67%) CAL gene (50%) (Table 1).

#### 3.2.6 Central America

In Mexico, a strain of the environmental clade (S. abietina) was reported as isolated also from insects. Identification to species level by the ITS region, β-tubulin gene, CAL gene, and other molecular methods with 100% each (Table 1).

#### 3.2.7 Oceania

In Tasmania, an isolates from environmental clade S. humicola, was identified from Dasyurus maculatus. The identification at the species level by the ITS region, β-tubulin gene and CAL gene with 100% each.

## 4 Discussion

Sporotrichosis is considered an emerging zoonosis with significant human and animal health implications. This mycosis usually causes nodules and ulcers on the skin and mucosa membranes, affecting lymph nodes and regional lymphatic vessels. It can even spread to other organs and cause severe forms that can lead to death, especially in cats and immunosuppressed humans (Gremião et al., 2020; Barros et al., 2011). In recent years, the evolution of this fungal disease has been gradually changing, not only in terms of frequency but also in modes of transmission, and geographic distribution. This can partly be explained by environmental changes, increased urbanization, poverty, and improved diagnoses (Montenegro et al., 2014).

The present study shows reports of 266 Sporothrix sp. isolates from animals worldwide for the period 2007–2021. Most isolates were reported from South America (*n* = 216 or 81%), followed by Asia (*n* = 28 or 10%), and Central America and Oceania (*n* = 1 or 0,37%) less frequently. After the description of new species of the genus Sporothrix, the identification of clinical isolates has been carried out worldwide, especially in regions where a large number of sporotrichosis cases occurs (Boechat et al., 2021), such as in southeastern Brazil, considered a zoonotic epidemic area of sporotrichosis (Gremião et al., 2017).

Phylogenetic analysis of Sporothrix species has traditionally been carried out using sequencing data of single or multiple conserved genes, mainly CHS, β-tubulin and the CAL gene. The latter is considered the reference standard for molecular identification of species of the genus Sporothrix (Marimon et al., 2007; New et al., 2019). The most commonly used molecular method in Europe, Asia, North, Central, and South America identified species by the CAL gene. On the African continent they were identified by the ITS region.

The species isolated with the higher number of samples and characterized by molecular tools, was S. brasiliensis (Brazil and Argentina) followed by S. schenckii (Argentina, Brazil, Japan, and Malaysia). These results corroborate studies that identified that zoonotic transmission by S. brasiliensis does not occur outside Brazil (Gremião et al., 2017), except in Argentina (Etchecopaz et al., 2019).

Fungal infections are often neglected (Seyedmousavi et al., 2015), and public health policies and strategic plans to prioritize these infections are lacking. Inadequate surveillance of fungal infections leads to unnoticed ocurrences, as seen in zoonotic sporotrichosis. Several reports have shown alarming concern about the occurrence of zoonotic sporotrichosis cases in non-endemic regions, such as the case of S. brasiliensis in Argentina, due to a potential transboundary expansion of the species. Despite regulations implemented for pet travel, a poor control of road transport can contribute to the spread of sporotrichosis in Brazil and worldwide (Gremião et al., 2020). Many studies have also identified that more than one species can be isolated within the same endemic area (Oliveira et al., 2011a, 2011b), as occurs in the city of Rio de Janeiro (Gremião et al., 2017).

Species of the environmental clade were isolated in all continents, and only in South America and Asia were species from the clinical clade isolated from animals. Due to this, we cannot ignore that even species belonging to the environmental clade present a relative risk of infection to animals. Corrêa-Moreira et al. (2020), demonstrated that the differences in virulence levels among these species might not be related to their taxonomic classification, considering that their results were quite heterogeneous when comparing "pathogenic" and "environmental" clade species in the experimental mice model, acting as an essential factor in the immunoregulatory mechanisms. For this reason, the species of the environmental clade can be virulent, possibly due to the interspecific variability that occurs between species of the genus Sporothrix. The second country with most feline isolated cases after Brazil was Malaysia. According to the study by Kano et al. (2015b), a genotype of S. schenckii that is adapting to the feline host may be occurring in Malaysia, similar to that reported for S. brasiliensis in Brazil, where an increase in the number of feline sporotrichosis cases caused by S. schenckii is occurring. Reports of feline cases have increased over the decades in many geographic areas in Brazil (Montenegro et al., 2014; Gremião et al., 2017). It was assumed that the thermal resistance exhibited by S. brasiliensis may be a vital adaptative mechanism of this fungus in cats (body temperature of 39°C) and may partially explain the success of infection of this species over other etiologic agents (Rodrigues et al., 2013a), such as S. globosa, which is more sensitive to temperatures above 35°C, but with case reports in humans (Oliveira et al., 2011b). This is easily observed in epidemiological studies, which showed that S. brasiliensis is feline host-dependent due to its occurrence in southern and southeastern Brazil (Rodrigues et al., 2013b). The increase in the number of cases in cats is often followed by an increase in the number of cases in humans, representing a serious public health problem. Although the increase in the number of cases of sporotrichosis in animals is proportional to the number of infections in humans, one of the limitations of this study is the scarcity of on cases of animal infection. This loss of data regarding the clinical aspects, drugs used and outcome of the infection, combined with the small number of studies identifying the fungus at the species level using molecular methodologies, is a major obstacle not only to our work, but also to the management of the disease.

For this reason, it is necessary to identify which species cause sporotrichosis, since each species has a specific virulence. Phenotypic and genotypic characteristics of different isolates within the genus Sporothrix were associated with their geographic distribution, virulence capacity, or clinical manifestation of sporotrichosis (Marimon et al., 2006; Oliveira et al., 2011b; Chakrabarti et al., 2015). However, there are few studies on animals, which are the main agents of human sporotrichosis, especially cat owners and veterinarians. The latter becoming a new risk group for acquiring sporotrichosis, due to the increased zoonotic potential, mainly from cats to humans in endemic regions of the disease. Nevertheless, in endemic areas, more people are at risk of acquiring zoonotic sporotrichosis due to the proximity between humans and cats (Gremião et al., 2015; Rodrigues et al., 2020). On the other hand, it is known that therapeutic measures for the treatment of animals, especially cats, take a long time and do not always respond well to treatment, with abandonment, recurrence of the lesion, or therapeutic failure, which may lead to death of the animal (Gremião et al., 2021).

When we refer to dogs, other important domestic animals with strict relationship with humans, only Italy (S. mexicana) and Brazil (S. brasiliensis, S. schenckii and S. luriei) samples of these animals were isolated in our study, as shown by Boechat et al. (2021) and Viana et al. (2018). Here, the dogs were also affected by sporotrichosis. However, the low fungal load observed in canine skin lesions appears to be a limiting factor for transmission compared to transmission in cats (Boechat et al., 2021; Viana et al., 2018). In the present study, all continents isolated samples from “other animals”, such as armadillos, insects, equines, and mice).

It should be noted that, although several authors report cases of sporotrichosis worldwide (Chakrabarti et al., 2015; Rodrigues et al., 2020), there are insufficient data on the molecular identification of species-level of the isolates obtained from animal. This has been one of the limitations of our study. Therefore, further studies on animal sporotrichosis and the molecular identification of species are needed. As an example, despite the estimated 22 million cats and 52 million dogs in Brazil (Junqueira and Galera, 2019), since 1988 only 244 canine cases (until 2014) and 5.113 feline cases (until 2017) were diagnosed and registered by the Evandro Chagas National Institute of Infectious Diseases (Rio de Janeiro) . We believe that this number of cases is underestimated. It is mainly because animal sporotrichosis (like human sporotrichosis) was subject to mandatory reporting only in some states or municipalities of Brazil. Additionally, molecular tools are not available in the routine diagnosis of human and animal cases worldwide as shown in this study. As a result, the number of animal cases diagnosed by molecular tools does not constitute a significant portion of the real cases in hyperendemic area of Rio de Janeiro.

The Brazilian picture of animal sporotrichosis can be extrapolated using worldwide occurrences, and in this context, as seen in the Covid-19 pandemic, with an increase in the number of new cases of fungi diseases by new or emerging fungus identified by molecular tools, we reinforce the need for more epidemiological studies using these tools. The One Health concept advocates the definition, identiffication, and monitoring of species potentially pathogenic to humans and animals.

Coordinated action between veterinarians, physicians, laboratory professionals, surveillance authorities and other health professionals, will ensure broader investigations and promote prevention, detection and assistance of human and animal cases (Gremião et al., 2020). Thus, epidemiological characterization of sporotrichosis for both animals and humans is necessary to implement health promotion, decrease sporotrichosis cases and confront this public health threat.

## 5 Conclusion

Our study confirmed a difficulty in obtaining the frequency of Sporothrix species, as seen in the molecular identification that has only been published in 13 countries. The most identified species were S. brasiliensis, isolated from cats in Brazil. And S. schenckii isolated from cats in Malaysia. This systematic review analyzed the geographic distribution of the species causing sporotrichosis in animals. We have shown the lack of studies in global areas and reinforced the need to use molecular tools to identify and monitor potential pathogens. This identification of Sporothrix at the species level by molecular tools in animals will strengthen the “One Health Concept”, which is a health promotion policy based on the integration between the health of humans, animals, and the environmental.

## CRediT authorship contribution statement

**Debora Salgado Morgado:** Methodology, Investigation, Formal analysis, Writing – original draft. **Rodolfo Castro:** Conceptualization, Methodology, Investigation, Formal analysis, Writing – review & editing. **Marcelo Ribeiro-Alves:** Conceptualization, Methodology, Investigation, Formal analysis, Writing – review & editing. **Danielly Corrêa-Moreira:** Supervision, Visualization, Writing – review & editing. **Julio Castro-Alves:** Investigation, Formal analysis, Writing – review & editing. **Sandro Antonio Pereira:** Writing – review & editing. **Rodrigo Caldas Menezes:** Supervision, Writing – review & editing. **Manoel Marques Evangelista Oliveira:** Conceptualization, Resources, Supervision, Project administration, Funding acquisition, Writing – review & editing.

## Declaration of Competing Interest

The authors declare that they have no known competing financial interests or personal relationships that could have appeared to influence the work reported in this paper.

## Appendix Supplementary materials

Image, application 1
