# Supplementary material for: Global distribution of animal sporotrichosis: A systematic review of Sporothrix sp. identified using molecular tools
Source: Curr Res Microb Sci. 2022 May 19;3:100140. doi: 10.1016/j.crmicr.2022.100140 (PMC9325896; doi:10.1016/j.crmicr.2022.100140)
Supplement: Supplementary file 1 [file mmc1.docx]

Supplementary table: Characteristics of the studies included in this review

| **Author** | **Origin of the isolates** | **Country of the study** | ***Sporothrix* species** | **Animal Species** | **Antifungal susceptibility (%)*** | | **Outcome*** | | |
| --- | --- | --- | --- | --- | --- | --- | --- | --- | --- |
|  |  |  |  |  | YES | NO | CURE | FAIL | N/A |
| Zhou *et al*., 2014 | USA | China | *S. brunneoviolacea* | Ant | ---- | 1 (100%) | ---- | ---- | 1 (100%) |
| de Beer *et al*., 2016 | USA | South Africa | *S. rossii* | Beetle | ---- | 1 (100%) | ---- | ---- | 1 (100%) |
| de Beer *et al.*, 2016 | Mexico | South Africa | *S. abietina* | Beetle | ---- | 1 (100%) | ---- | ---- | 1 (100%) |
| Rodrigues *et al.,* 2013; Córdoba *et al*., 2018 | Argentina | Brazil  Argentina | *S. brasiliensis* | Cat | 1  (50%) | 1  (50%) | ---- | ---- | 1  (50%) |
| Córdoba *et al.,* 2018 | Argentina | Argentina | *S. schenckii* | Horse, Mouse | 1 (100%) |  | ---- | ---- | 1  (100%) |
| Oliveira *et al.,* 2011; 2013; Castro *et al*., 2013; Fernandes *et al*., 2013; Rodrigues *et al*., 2013; 2014a; 2014b; 2014c; 2015; 2016; Sasak *et al.,* 2014; Teixeira *et al.,* 2014; Araujo *et al.,* 2015; Zhang *et al.,* 2015; Boechat *et al*., 2018; Córdoba *et al.*, 2018; Boechat *et al.,* 2020; Macêdo-Sales *et al*., 2020; Spanamberg *et al*., 2020; | Brazil | Brazil  China  Argentina | *S. brasiliensis* | Cat | 5  (26%) | 14 (74%) | 2  (10%) | 2  (10%) | 15 (79%) |
| Rodrigues *et al.,* 2013; Zhang *et al.,* 2015; Boechat *et al*., 2020 | Brazil | Brazil  China | *S. brasiliensis* | Dog | 1  (33%) | 2  (67%) | 1  (33%) | 1  (33%) | 2  (67%) |
| Oliveira *et al.*, 2011 | Brazil | Brazil | *S. luriei* | Dog | 1 (100%) | ---- | ---- | ---- | 1  (100%) |
| Oliveira *et al.*, 2011; Rodrigues *et al*., 2014a; 2014b; Sasak *et al.,* 2014 | Brazil | Brazil | *S. pallida* | Cat | 2  (50%) | 2  (50%) | ---- | ---- | 4  (100%) |
| Rodrigues *et al*., 2013; 2014a; 2014c; Zhang *et al*., 2015 | Brazil | Brazil  China | *S. schenckii* | Cat | ---- | 4 (100%) | ---- | ---- | 4  (100%) |
| Stopiglia *et al.*, 2014; Boechat *et al.*, 2020 | Brazil | Brazil | *S. schenckii* | Dog | 2 (100%) | ---- | 1  (50%) | 1  (50%) | 1  (50%) |
| de Beer *et al.*, 2016 | Spain | South Africa | *O. stenoceras* | Weevil | ---- | 1 (100%) | ---- | ---- | 1  (100%) |
| de Beer *et al.*, 2016 | Spain | South Africa | *S. cantabriensis* | Beetle | ---- | 1 (100%) | ---- | ---- | 1  (100%) |
| de Beer *et al*., 2016 | Spain | South Africa | *S. euskadiensis* | Beetle | ---- | 1 (100%) | ---- | ---- | 1  (100%) |
| de Beer *et al.*, 2016 | Spain | South Africa | *S. nebularis* | Beetle | ---- | 1 (100%) | ---- | ---- | 1  (100%) |
| Zhang *et al*., 2015 | Italy | China | *S. mexicana* | Dog | ---- | 1 (100%) | ---- | ---- | 1  (100%) |
| Marimon *et al*., 2007; 2008; Romeo *et al.,* 2011; Rodrigues *et al*., 2013; 2014c; Zhang *et al.,* 2015 | Germany | Spain  Italy  Brazil  China | *S. pallida* | Termite | ---- | 6 (100%) | ---- | ---- | 6  (100%) |
| Makri *et al.,* 2020 | UK | UK | *S. humicola* | Cat | ---- | 1 (100%) | 1 (100%) |  |  |
| de Beer e*t al.*, 2016 | South Africa | South Africa | *S. aurorae* | Beetle | ---- | 1 (100%) | ---- | ---- | 1  (100%) |
| de Beer *et al*., 2016 | South Africa | South Africa | *S. gemella* | Mite | ---- | 1 (100%) | ---- | ---- | 1  (100%) |
| Zhang *et a*l., 2015 | South Africa | China | *S. gemellus* | Mite | ---- | 1 (100%) | ---- | ---- | 1  (100%) |
| Zhou *et al.,* 2014; Zhang *et al.,* 2015; de Beer *et al*., 2016 | South Africa | China | *S. variecibatus* | Mite | ---- | 3 (100%) | ---- | ---- | 3  (100%) |
| Azam *et al.,* 2019; Kano *et al*., 2015 | Malaysia | Malaysia | *S. schenckii* | Cat | ---- | 2 (100%) | ---- | ---- | 2  (100%) |
| Kano *et al.,* 2013; Kano *et al*., 2015 | Japan | Japan | *S. globosa* | Cat | ---- | 2 (100%) | ---- | ---- | 2  (100%) |
| Nesseler *et al.,* 2019 | Tasmania | Germany | *S. humicola* | Tiger-quoll | ---- | 1 (100%) | ---- | ---- | 2  (100%) |

* Percentage of studies in which were performed antifungal susceptibility of the samples and/or relate the outcome of the animal infection. N/A (not applicable) = have no mention regarding the outcome.
